# Machine learning for the real-time assessment of left ventricular ejection fraction in critically ill patients: a bedside evaluation by novices and experts in echocardiography

**DOI:** 10.1186/s13054-022-04269-6

**Published:** 2022-12-14

**Authors:** Rita Varudo, Filipe A. Gonzalez, João Leote, Cristina Martins, Jacobo Bacariza, Antero Fernandes, Frederic Michard

**Affiliations:** 1 Intensive Care Department, Hospital Garcia de Orta, Almada, Portugal; 2 Faculdade de Medicina da Universidade de Lisboa, Lisbon, Portugal; 3 Faculdade de Ciencias da Saude da Universidade da Beira Interior, Covilha, Portugal; 4 MiCo, Vallamand, Switzerland

**Keywords:** Echocardiography, Left ventricular ejection fraction, Artificial intelligence, Machine learning, Point of care ultrasound, Novice

## Abstract

**Background:**

Machine learning algorithms have recently been developed to enable the automatic and real-time echocardiographic assessment of left ventricular ejection fraction (LVEF) and have not been evaluated in critically ill patients.

**Methods:**

Real-time LVEF was prospectively measured in 95 ICU patients with a machine learning algorithm installed on a cart-based ultrasound system. Real-time measurements taken by novices (LVEF_Nov_) and by experts (LVEF_Exp_) were compared with LVEF reference measurements (LVEF_Ref_) taken manually by echo experts.

**Results:**

LVEF_Ref_ ranged from 26 to 80% (mean 54 ± 12%), and the reproducibility of measurements was 9 ± 6%. Thirty patients (32%) had a LVEF_Ref_ < 50% (left ventricular systolic dysfunction). Real-time LVEF_Exp_ and LVEF_Nov_ measurements ranged from 31 to 68% (mean 54 ± 10%) and from 28 to 70% (mean 54 ± 9%), respectively. The reproducibility of measurements was comparable for LVEF_Exp_ (5 ± 4%) and for LVEF_Nov_ (6 ± 5%) and significantly better than for reference measurements (*p* < 0.001). We observed a strong relationship between LVEF_Ref_ and both real-time LVEF_Exp_ (*r* = 0.86, *p* < 0.001) and LVEF_Nov_ (*r* = 0.81, *p* < 0.001). The average difference (bias) between real time and reference measurements was 0 ± 6% for LVEF_Exp_ and 0 ± 7% for LVEF_Nov_. The sensitivity to detect systolic dysfunction was 70% for real-time LVEF_Exp_ and 73% for LVEF_Nov_. The specificity to detect systolic dysfunction was 98% both for LVEF_Exp_ and LVEF_Nov_.

**Conclusion:**

Machine learning-enabled real-time measurements of LVEF were strongly correlated with manual measurements obtained by experts. The accuracy of real-time LVEF measurements was excellent, and the precision was fair. The reproducibility of LVEF measurements was better with the machine learning system. The specificity to detect left ventricular dysfunction was excellent both for experts and for novices, whereas the sensitivity could be improved.

*Trial registration*: NCT05336448. Retrospectively registered on April 19, 2022.

**Supplementary Information:**

The online version contains supplementary material available at 10.1186/s13054-022-04269-6.

## Introduction

The assessment of left ventricular ejection fraction (LVEF) is part of the point of care echocardiographic evaluation of critically ill patients [1–3]. It has the disadvantage of being time-consuming and operator dependent. Machine learning algorithms have recently been developed to facilitate, automate, and decrease the variability of echocardiographic measurements [4–7]. Several algorithms have been designed specifically for the real-time assessment of LVEF [8–10]. They have been trained to recognize specific ultrasound images, enable instantaneous image quality control, and measure LVEF automatically in just a few seconds. However, clinical validation studies remain scarce and have been done in ambulatory cardiac patients [8–10].

In critically ill patients, we compared real-time LVEF measurements taken with a new machine learning algorithm to reference manual measurements taken by experts in echocardiography.

## Methods

We prospectively studied critically ill patients who required an echocardiographic evaluation during their ICU stay and in whom it was possible to obtain transthoracic images enabling a manual and quantitative evaluation of left ventricular systolic function. Real-time LVEF measurements were taken with a machine learning algorithm (Real-Time EF, GE Healthcare, Chicago, USA) installed on a cart-based ultrasound system (Venue, GE Healthcare). The real-time LVEF software is a neural network algorithm which has been trained with thousands of cardiac images to automatically detect the 4-chamber view of the heart, locate landmarks on the left ventricular wall and detect end-diastolic and end-systolic times from the mitral valve motion. Once the endocardial border is detected, the algorithm provides immediate user feedback regarding image quality using color-coding. When image quality is considered acceptable (green or yellow endocardial border displayed on screen), left ventricular volumes are automatically estimated from the single-plane Simpson disk method, enabling LVEF calculation from real-time end-diastolic and end-systolic volumes.

Real-time LVEF measurements obtained by a novice (LVEF_Nov_) and by an expert (LVEF_Exp_) were compared with LVEF measurements taken manually by an expert in critical care echocardiography (LVEF_Ref_). Seven novices (all residents in our department and beginners in echocardiography) and two experts (senior intensivists with the European Diploma in Advanced Critical Care Echocardiography) participated in data collection. Measurements taken in triplicate were averaged for comparisons, and the intra-operator reproducibility was assessed by calculating the coefficient of variation (standard deviation divided by the mean) expressed as a percentage.

The quality of echo images was classified as good, fair, or poor by the experts, and as green (optimal), yellow (acceptable), or red (not acceptable for real-time LVEF measurements) by the machine learning algorithm.

Results are expressed as mean ± standard deviation (SD). Agreement between real-time and reference LVEF measurements was tested using the Bland–Altman method. Statistical comparisons were made with a *t*-test. A *p* value < 0.05 was considered statistically significant.

## Results

We prospectively enrolled 95 patients (mean age 60 ± 17 yr) over a 9-month period. Most patients were admitted for medical reasons and 32 (34%) were mechanically ventilated at the time of the ultrasound evaluation (Additional file 1: Table S1). Reference LVEF ranged from 26 to 80% (mean 54 ± 12%) and the reproducibility of manual measurements was 9 ± 6%. Thirty patients (32%) had a LVEF_Ref_ < 50% (left ventricular systolic dysfunction).

**Fig. 1:**
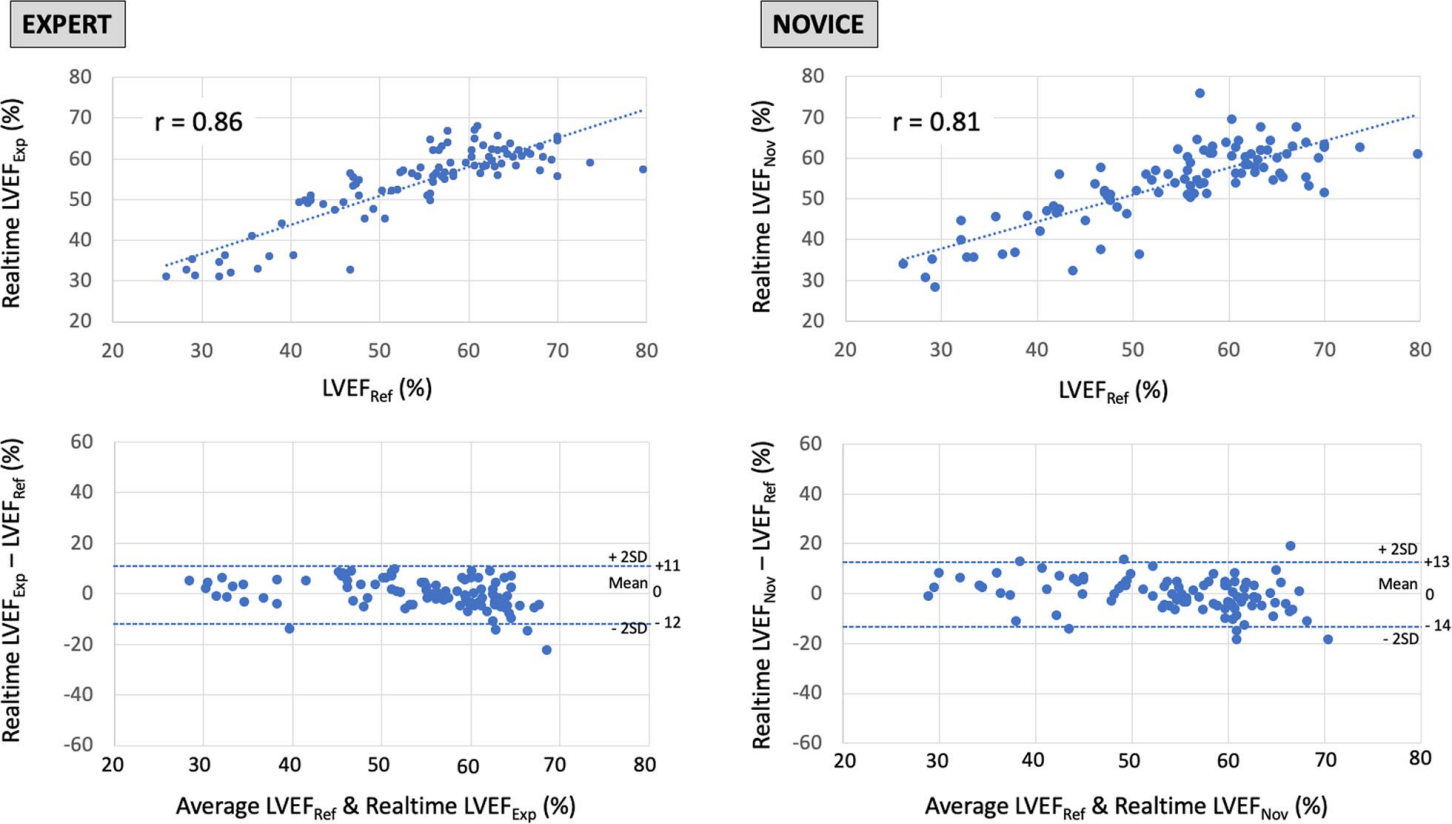
Correlation and Bland and Altman comparison between reference left ventricular ejection fraction measurements taken by experts (LVEF_Ref_) and real-time measurements taken with a machine learning algorithm. Left: real-time measurements taken by experts (LVEF_Exp_), right: real-time measurements taken by novices (LVEF_Nov_)

Real-time LVEF_Exp_ ranged from 31 to 68% (mean 54 ± 10%). We observed a strong relationship (*r* = 0.86, *p* < 0.001) between reference and real-time LVEF_Exp_ (Fig. 1). The average difference (bias) between real-time LVEF_Exp_ and reference LVEF was 0 ± 6% with 95% limits of agreement of − 12 to + 11% (Fig. 1). The intra-operator reproducibility of measurements was better for real-time LVEF_Exp_ than for reference manual measurements (5 ± 4% vs. 9 ± 6%, *p* < 0.001). The sensitivity and specificity of real-time LVEF_Exp_ to detect systolic dysfunction were 70% and 98%, respectively.

Real-time LVEF_Nov_ ranged from 28 to 70% (mean 54 ± 9%). We observed a strong relationship (*r* = 0.81, *p* < 0.001) between LVEF_Ref_ and real-time LVEF_Nov_ (Fig. 1). The average difference (bias) between real-time LVEF_Nov_ and LVEF_Ref_ was 0 ± 7% with 95% limits of agreement of − 14 to + 13% (Fig. 1). The intra-operator reproducibility of measurements was better for real-time LVEF_Nov_ than for reference manual measurements (6 ± 5% vs. 9 ± 6%, *p* < 0.001). The sensitivity and specificity of real-time LVEF_Nov_ to detect systolic dysfunction were 73% and 98%, respectively.

According to experts’ judgement, the quality of echo images was good, fair, and poor in 41, 43, and 11 patients, respectively. The average difference (bias) between real-time and reference LVEF measurements was comparable when images were of good quality (*n* = 41) and of fair or poor quality (*n* = 54), both for experts and novices (Table 1). And results did not change significantly after excluding the 11 patients with poor image quality (Table 1).

**Table 1:** Main results in subgroups based on image quality

Image quality	Good	Good	Good and fair	Good and fair	Fair and poor	Fair and poor	Green-flagged	Green-flagged
Measurement	Manual	Manual	Manual	Manual	Manual	Manual	ML	ML
Operator	Expert	Novice	Expert	Novice	Expert	Novice	Expert	Novice
Patients, n	41	41	84	84	54	54	80	80
Reproducibility	5 ± 4%	6 ± 4%	5 ± 4%	6 ± 5%	5 ± 4%	5 ± 5%	5 ± 4%	6 ± 5%
Correlation coef.	*r* = 0.86	*r* = 0.78	*r* = 0.85	*r* = 0.81	*r* = 0.86	*r* = 0.83	*r* = 0.86	*r* = 0.82
Bias (mean ± SD)	0 ± 6%	0 ± 7%	0 ± 6%	0 ± 7%	− 1 ± 6%	− 1 ± 6%	0 ± 6%	0 ± 7%
Sensitivity	–	–	65%	73%	–	–	65%	69%
Specificity	–	–	100%	100%	–	–	100%	100%

ML = Machine learning, coef. = coefficient. Sensitivity and specificity were not calculated for small subgroups

According to the machine learning algorithm, the quality of echo images was green, yellow, and red flagged in 80, 15 and 0 patients, respectively. Results did not change significantly after excluding the 15 patients in whom images were non-optimal/yellow flagged (Table 1).

The average difference (bias) between real-time and reference LVEF measurements was slightly higher in mechanically ventilated (*n* = 32) than in non-mechanically ventilated patients (*n* = 63), both for experts (− 2 ± 7% vs. 0 ± 5%) and novices (− 1 ± 8% vs. 0 ± 6%). However, observed differences did not reach statistical significance.

## Discussion

An increasing number of anesthesiologists and intensivists have been trained to perform qualitative echocardiographic assessments [1–3]. However, quantitative evaluations remain challenging for many, particularly for novices. In the present study, we tested an artificial intelligence-enabled tool specifically designed to facilitate and automatize the bedside measurements of LVEF. Our findings suggest that this tool enables a clinically acceptable estimation of LVEF when compared to manual measurements. They also suggest that the real-time LVEF tool enables novices to assess LVEF with a better reproducibility than what experts can achieve manually.

Several machine learning algorithms have been designed to assess LVEF from a parasternal long axis view or from an apical 2 or 4-chamber view [8–10]. Comparison studies published so far yielded promising results. Indeed, close correlations and good agreements have been reported between LVEF measurements taken by skilled operators and by machine learning algorithms, particularly when the algorithm detects and analyze the apical 4-chamber view [9, 10]. However, clinical validation studies remain scarce and have been done in ambulatory cardiac patients. Our study appears to be the first evaluation done in critically ill patients in whom transthoracic echocardiography is often challenging, in particular when patients are mechanically ventilated. Our findings suggest that the real-time LVEF algorithm may help clinicians, including beginners in echocardiography, to accurately measure LVEF in just a few seconds. Such a tool may contribute to further increase the adoption of point of care echocardiographic evaluations in critically ill patients.

Our study has limitations. Because ultrasound evaluations are time-consuming, we studied hemodynamically stable patients to ensure comparability between measurements taken at each step of the evaluation (LVEF measurements were first taken by a trainee, then by an expert both manually and with the automatic method). Also, we did not assess the ability of the new real-time LVEF method to track changes in LVEF. A small number of patients had a severely impaired left ventricular systolic function (LVEF_Ref_ < 30%, *n* = 4) or a hyperkinetic ventricle (LVEF_Ref_ > 70%, *n* = 2). Therefore, future studies will need to assess the clinical value of the real-time LVEF algorithm during hemodynamic instability, in patients with a very low or supranormal LVEF, and during therapeutic interventions (e.g., inotropic stimulation) known to induce significant changes in systolic function.

## Conclusion

Machine learning-enabled real-time measurements of LVEF were strongly correlated with manual measurements obtained by experts. The accuracy of real-time LVEF measurements was excellent, and the precision was fair. The reproducibility of LVEF measurements was better with the machine learning system, including for novices. The specificity to detect left ventricular systolic dysfunction was excellent both for experts and novices, whereas the sensitivity could be improved. Studies are needed to confirm our findings in mechanically ventilated patients with cardiogenic shock or hyperdynamic states.

## Supplementary Information

Additional file 1: Table S1 Main characteristics of the study population.

## Data Availability

The datasets used and/or analyzed during the current study are available from the corresponding author on reasonable request.

## Abbreviations

LVEF: Left ventricular ejection fraction
LVEF_Ref_: Reference left ventricular ejection fraction
LVEF_Exp_: Real-time left ventricular ejection fraction measured by experts
LVEF_Nov_: Real-time left ventricular ejection fraction measured by novices
