# Supplementary material for: Machine learning for the real-time assessment of left ventricular ejection fraction in critically ill patients: a bedside evaluation by novices and experts in echocardiography
Source: Crit Care. 2022 Dec 14;26:386. doi: 10.1186/s13054-022-04269-6 (PMC9749290; doi:10.1186/s13054-022-04269-6)
Supplement: Supplementary file 1 — Additional file 1: Table S1 Main characteristics of the study population. [file 13054_2022_4269_MOESM1_ESM.docx]

**Additional file**

**Table S1. Main characteristics of the study population**

| Gender, F/M | 31/64 |
| --- | --- |
| Age, yr | 60 ± 17 |
| Body Mass Index, kg/m^2^ | 27 ± 6 |
| **COMORBIDITIES** |  |
| Chronic hypertension, n (%) | 58 (61%) |
| Diabetes, n (%) | 24 (25%) |
| Coronary artery disease, n (%) | 12 (13%) |
| Chronic renal failure, n (%) | 9 (9%) |
| Chronic liver disease, n (%) | 5 (5%) |
| **MAIN REASON FOR ICU ADMISSION** |  |
| Neurologic disease, n (%) | 28 (29%) |
| Cardiovascular disease, n (%) | 13 (14%) |
| Respiratory disease, n (%) | 12 (13%) |
| Postoperative surveillance, n (%) | 11 (12%) |
| Other, n (%) | 31 (33%) |
| **SCORES AT ICU ADMISSION** |  |
| APACHE II | 14 ± 10 |
| SAPS II | 34 ± 19 |
| SOFA | 4.5 ± 3.7 |
| **AT THE TIME OF ECHO EVALUATION** |  |
| Heart rate, bpm | 77 ± 15 |
| Mean arterial pressure, mmHg | 86 ± 16 |
| Mechanical ventilation, n (%) | 32 (34%) |
| Vasopressor support, n (%) | 29 (31%) |
| Inotropic support, n (%) | 5 (5%) |
